# Supplementary figures and images for: Differential plasmacytoid dendritic cell phenotype and type I Interferon response in asymptomatic and severe COVID-19 infection
Source: PLoS Pathog. 2021 Sep 2;17(9):e1009878. doi: 10.1371/journal.ppat.1009878 (PMC8412261; doi:10.1371/journal.ppat.1009878)

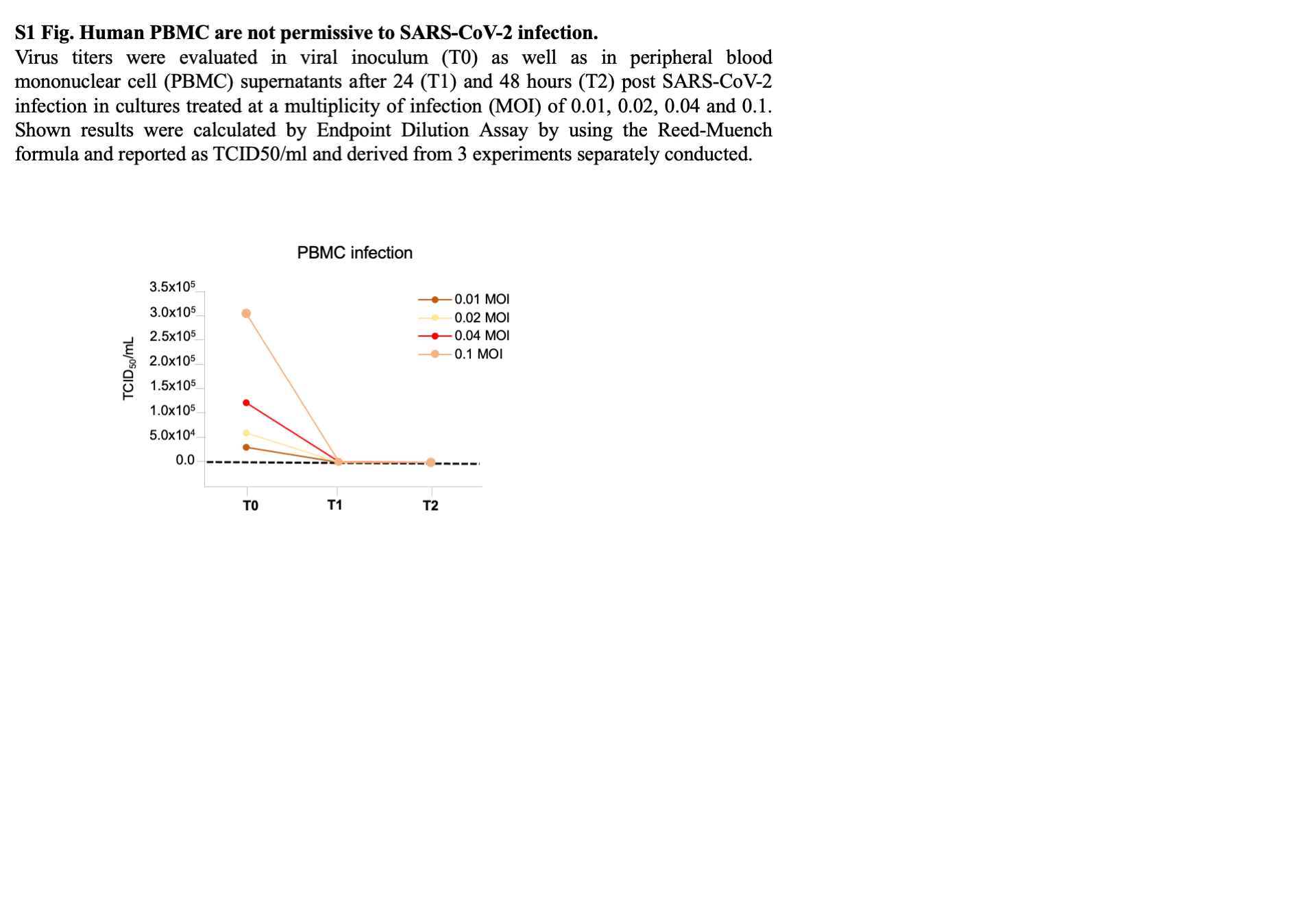

Supplement: S1 Fig — Virus titers were evaluated in viral inoculum (T0) as well as in peripheral blood mononuclear cell (PBMC) supernatants after 24 (T1) and 48 hours (T2) post SARS-CoV-2 infection in cultures treated at a multiplicity of infection (MOI) of 0.01, 0.02, 0.04 and 0.1. Shown results were calculated by Endpoint Dilution Assay by using the Reed-Muench formula and reported as TCID50/ml and derived from 3 experiments separately conducted. (TIFF) [file ppat.1009878.s001.tiff]

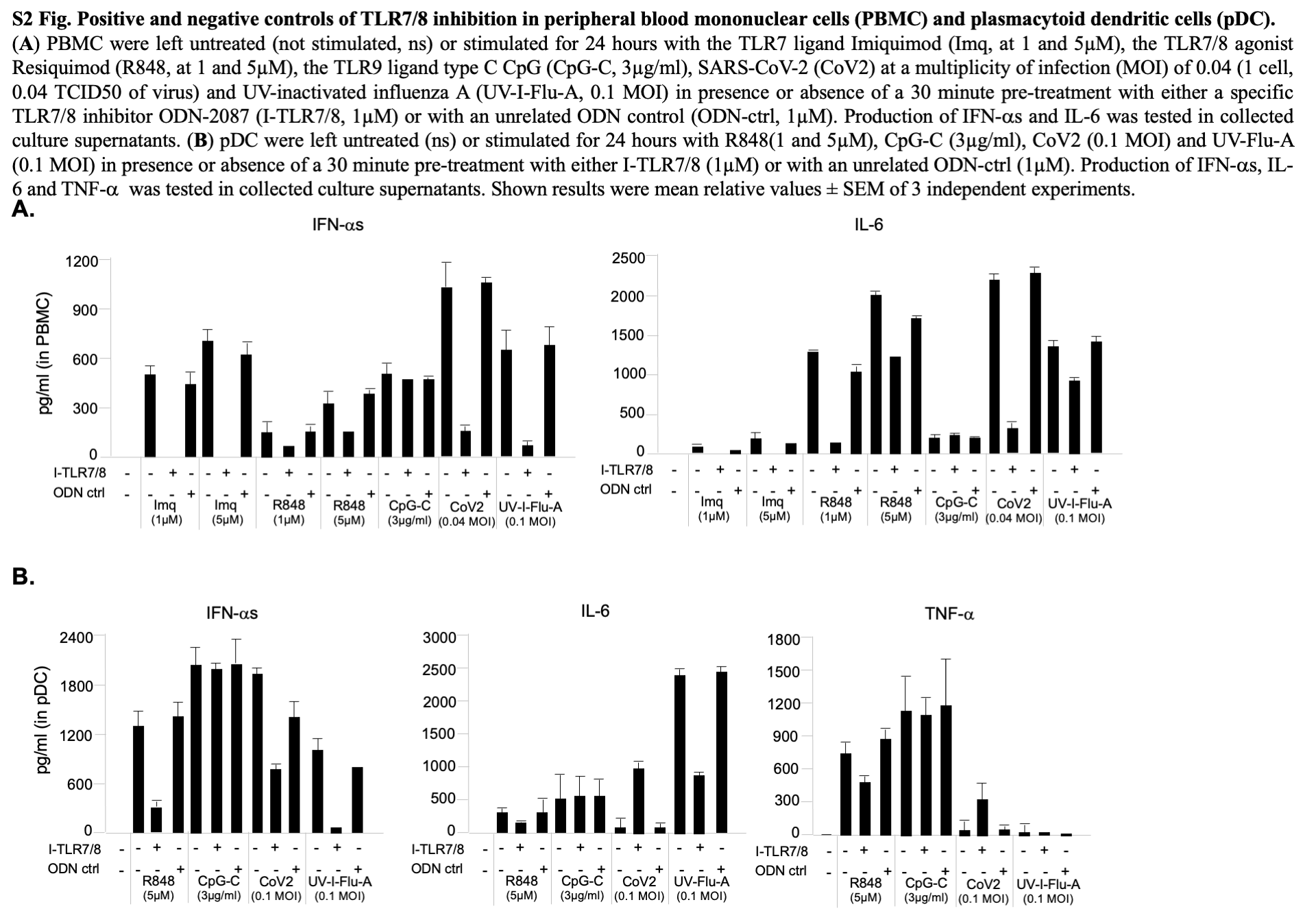

Supplement: S2 Fig — (A) PBMC were left untreated (not stimulated, ns) or stimulated for 24 hours with the TLR7 ligand Imiquimod (Imq, at 1 and 5μM), the TLR7/8 agonist Resiquimod (R848, at 1 and 5μM), the TLR9 ligand type C CpG (CpG-C, 3μg/ml), SARS-CoV-2 (CoV2) at a multiplicity of infection (MOI) of 0.04 (1 cell, 0.04 TCID50 of virus) and UV-inactivated influenza A (UV-I-Flu-A, 0.1 MOI) in presence or absence of a 30 minute pre-treatment with either a specific TLR7/8 inhibitor ODN-2087 (I-TLR7/8, 1μM) or an unrelated ODN control (ODN-ctrl, 1μM). Production of IFN-αs and IL-6 was tested in collected culture supernatants. (B) pDC were left untreated (ns) or stimulated for 24 hours with R848 (1 and 5μM), CpG-C (3μg/ml), CoV2 (0.1 MOI) and UV-Flu-A (0.1 MOI) in presence or absence of a 30 minute pre-treatment with either I-TLR7/8 (1μM) or with an unrelated ODN-ctrl (1μM). Production of IFN-αs, IL-6 and TNF-α was tested in collected culture supernatants. Shown results were mean relative values ± SEM of 3 independent experiments. (TIFF) [file ppat.1009878.s002.tiff]

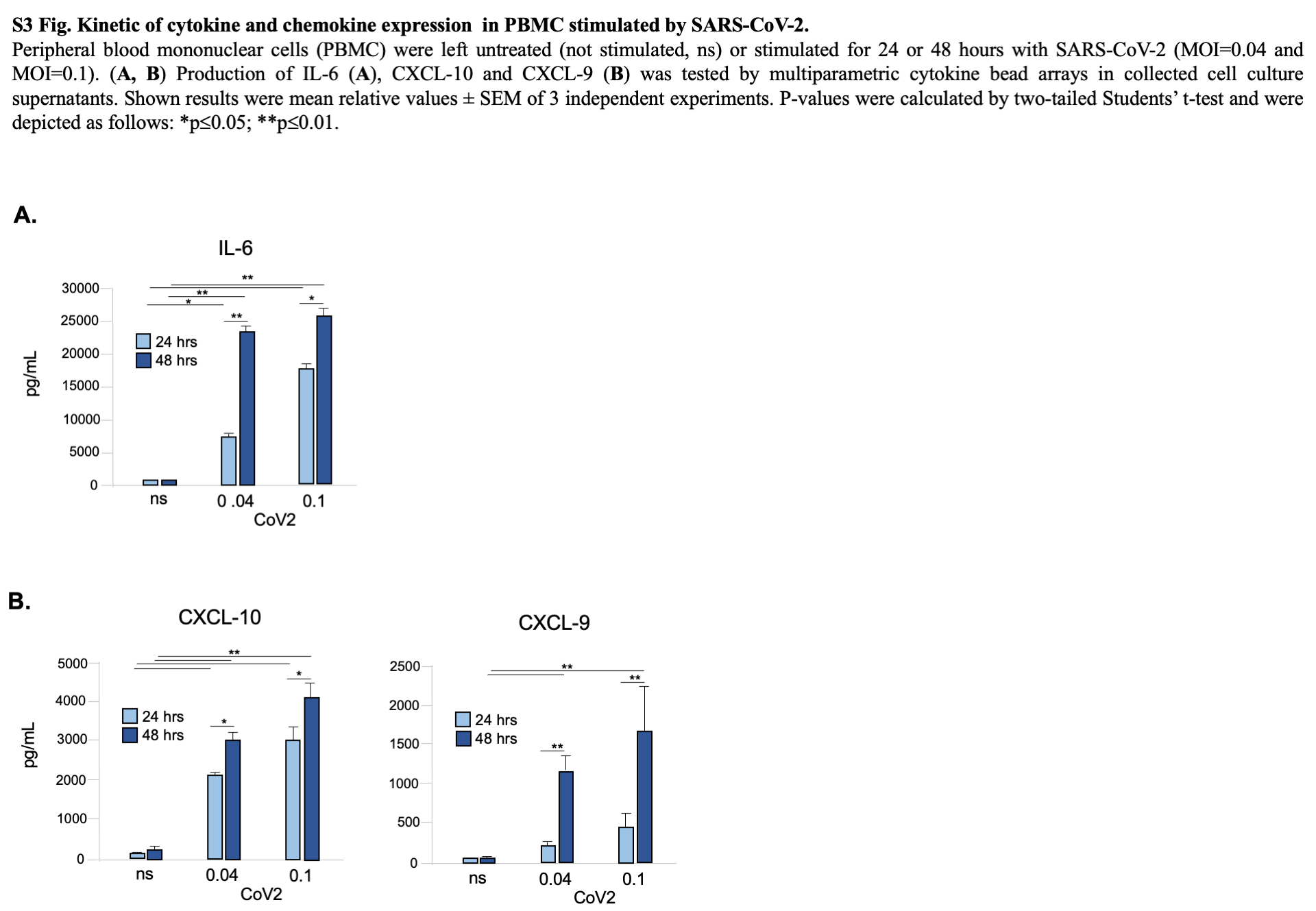

Supplement: S3 Fig — Peripheral blood mononuclear cells (PBMC) were left untreated (not stimulated, ns) or stimulated for 24 or 48 hours with SARS-CoV-2 (MOI = 0.04 and MOI = 0.1). (A, B) Production of IL-6 (A), and CXCL-10 and CXCL-9 (B) was tested by multiparametric cytokine bead arrays in collected cell culture supernatants. Shown results were mean relative values ± SEM of 3 independent experiments. P-values were calculated by two-tailed Students’ t-test and were depicted as follows: *p≤0.05; **p≤0.01. (TIFF) [file ppat.1009878.s003.tiff]

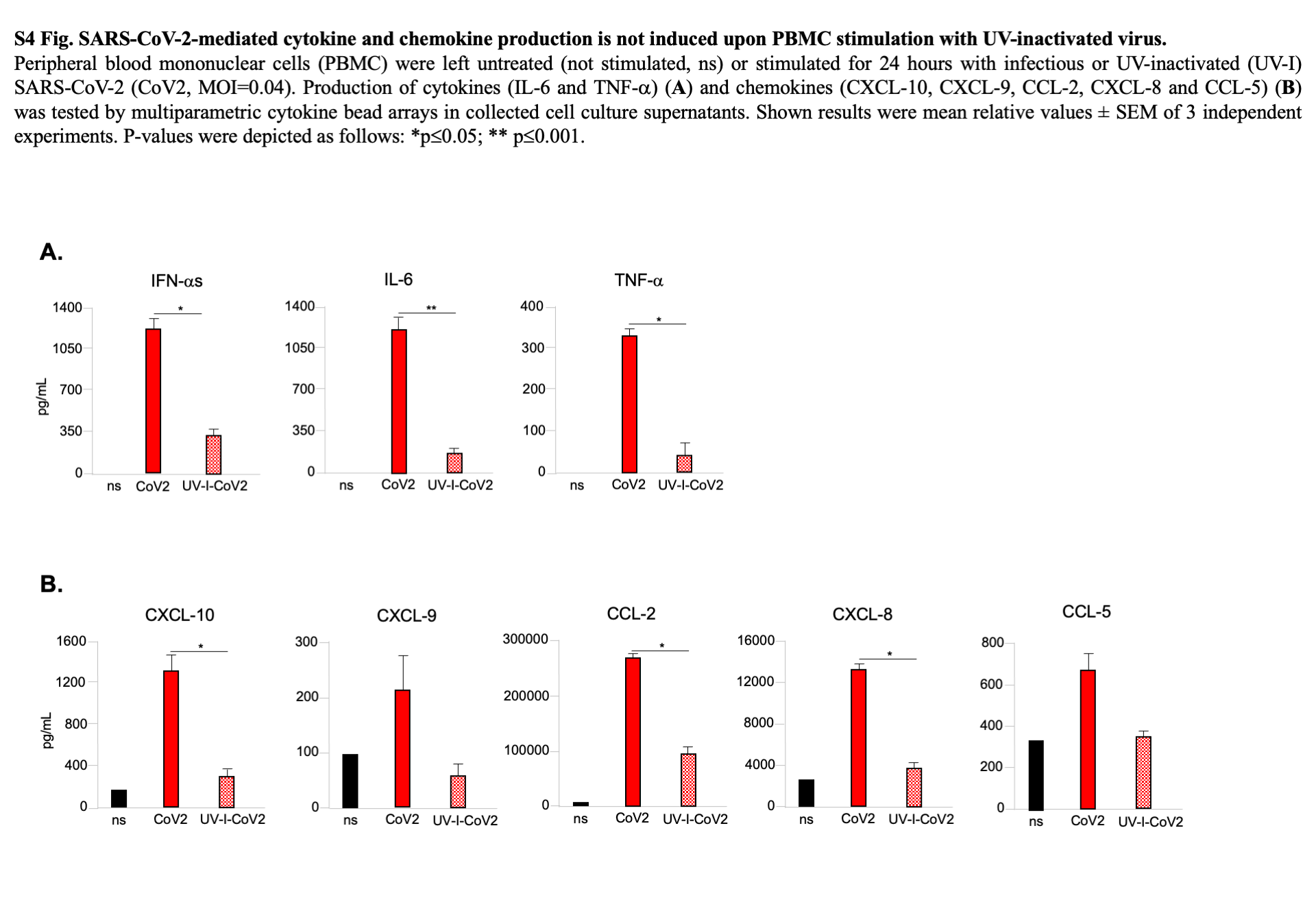

Supplement: S4 Fig — Peripheral blood mononuclear cells (PBMC) were left untreated (not stimulated, ns) or stimulated for 24 hours with infectious or UV-inactivated (UV-I) SARS-CoV-2 (CoV2, MOI = 0.04). Production of cytokines (IL-6 and TNF-α) (A) and chemokines (CXCL-10, CXCL-9, CCL-2, CXCL-8 and CCL-5) (B) was tested by multiparametric cytokine bead arrays in collected cell culture supernatants. Shown results were mean relative values ± SEM of 3 independent experiments. P-values were depicted as follows: *p≤0.05; ** p≤0.001. (TIFF) [file ppat.1009878.s004.tiff]

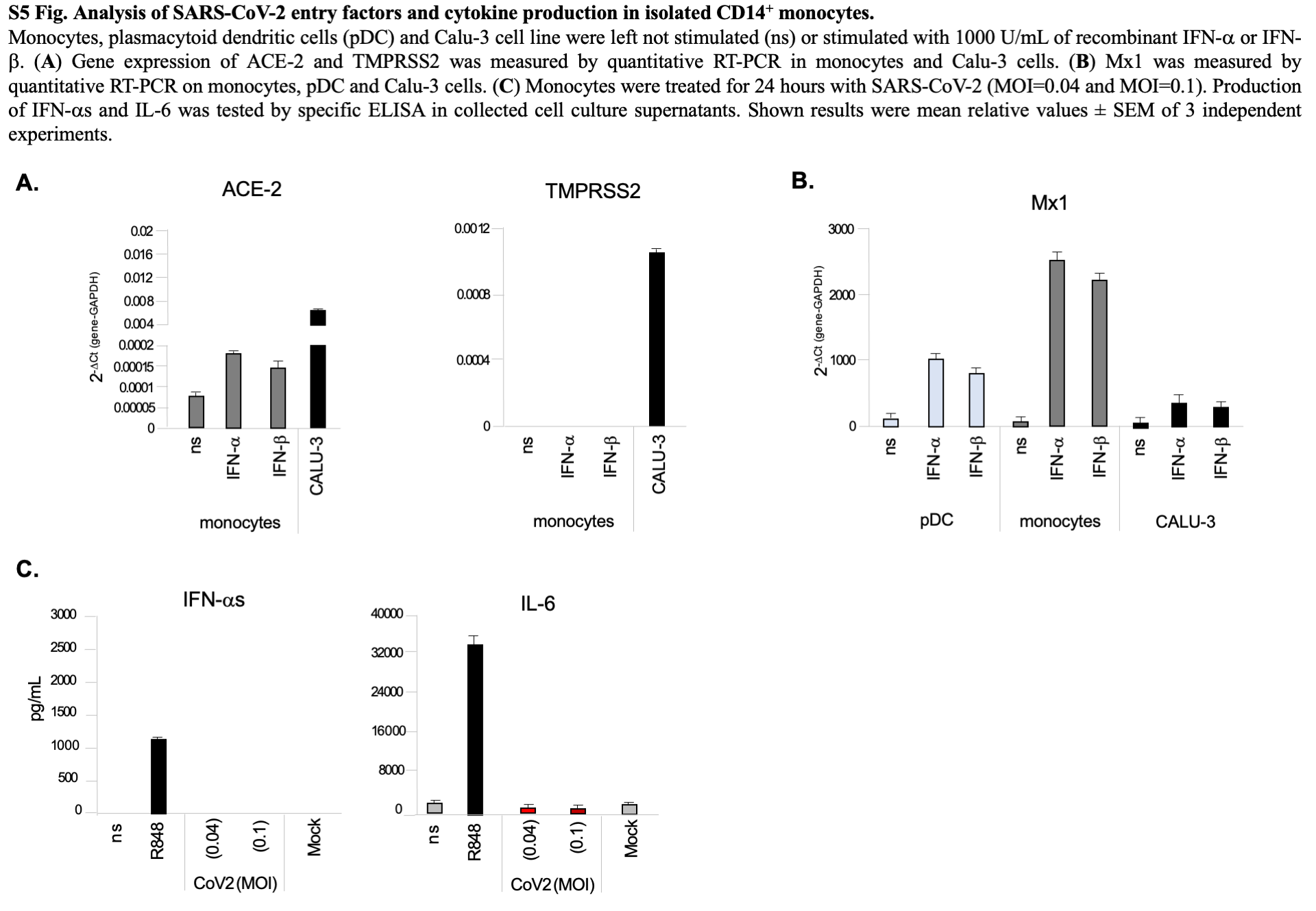

Supplement: S5 Fig — Monocytes, plasmacytoid dendritic cells (pDC) and Calu-3 cell line were left not stimulated (ns) or stimulated with 1000 U/mL of recombinant IFN-α or IFN-β. (A) Gene expression of ACE-2 and TMPRSS2 was measured by quantitative RT-PCR in monocytes and Calu-3 cells. (B) Mx1 was measured by quantitative RT-PCR on monocytes, pDC and Calu-3 cells. (C) Monocytes were treated for 24 hours with SARS-CoV-2 (MOI = 0.04 and MOI = 0.1). Production of IFN-αs and IL-6 was tested by specific ELISA in collected cell culture supernatants. Shown results were mean relative values ± SEM of 3 independent experiments. (TIFF) [file ppat.1009878.s005.tiff]

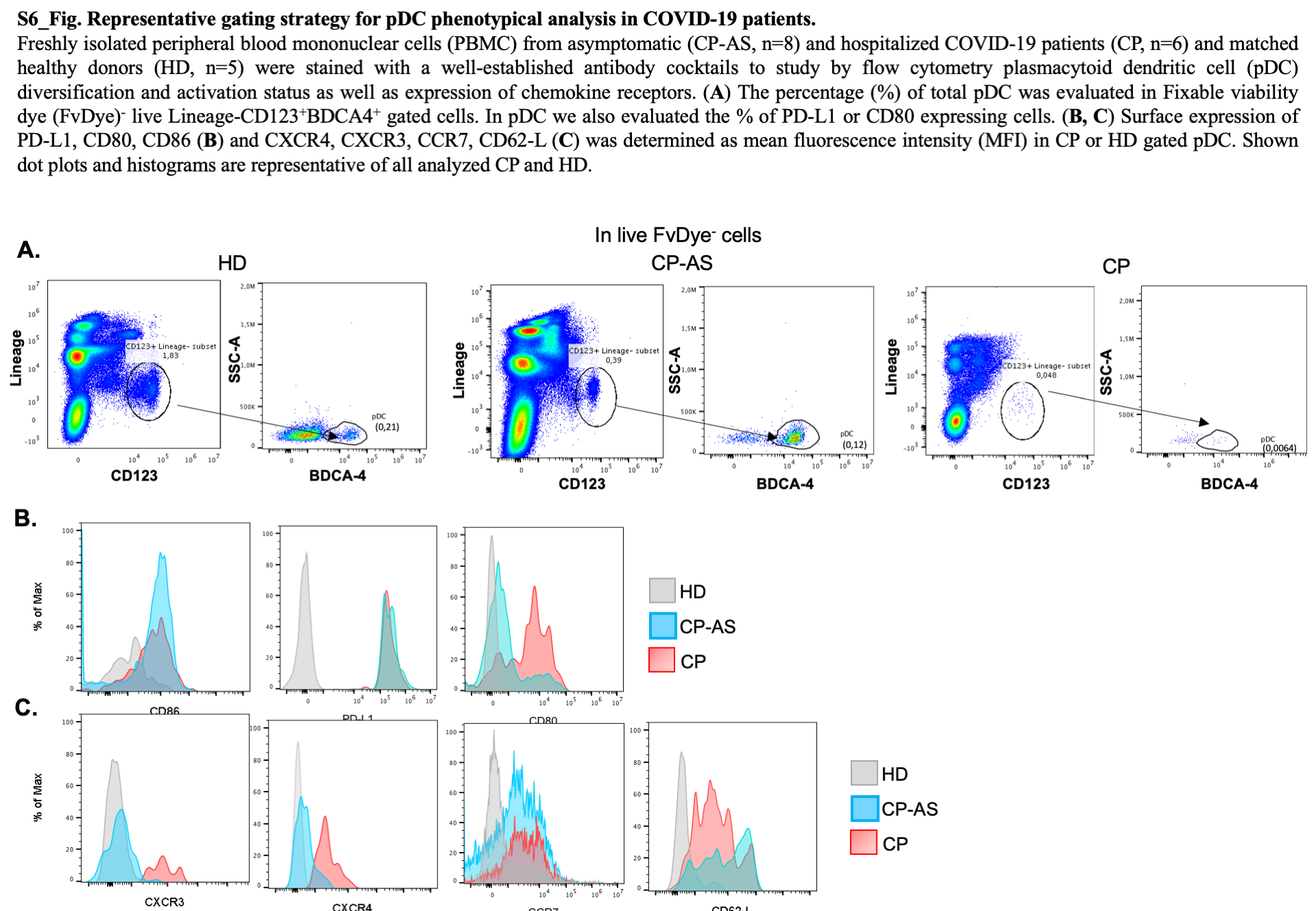

Supplement: S6 Fig — Freshly isolated peripheral blood mononuclear cells (PBMC) from asymptomatic (CP-AS, n = 8) and hospitalized COVID-19 patients (CP, n = 6) and matched healthy donors (HD, n = 5) were stained with a well-established antibody cocktails to study by flow cytometry plasmacytoid dendritic cell (pDC) diversification and activation status as well as expression of chemokine receptors. (A) The percentage (%) of total pDC was evaluated in Fixable viability dye (FvDye)- live Lineage-CD123+BDCA4+ gated cells. In pDC we also evaluated the % of PD-L1 or CD80 expressing cells. (B, C) Surface expression of PD-L1, CD80, CD86 (B) and CXCR4, CXCR3, CCR7, CD62-L (C) was determined as mean fluorescence intensity (MFI) in CP or HD gated pDC. Shown dot plots and histograms are representative of all analyzed CP and HD. (TIFF) [file ppat.1009878.s006.tiff]

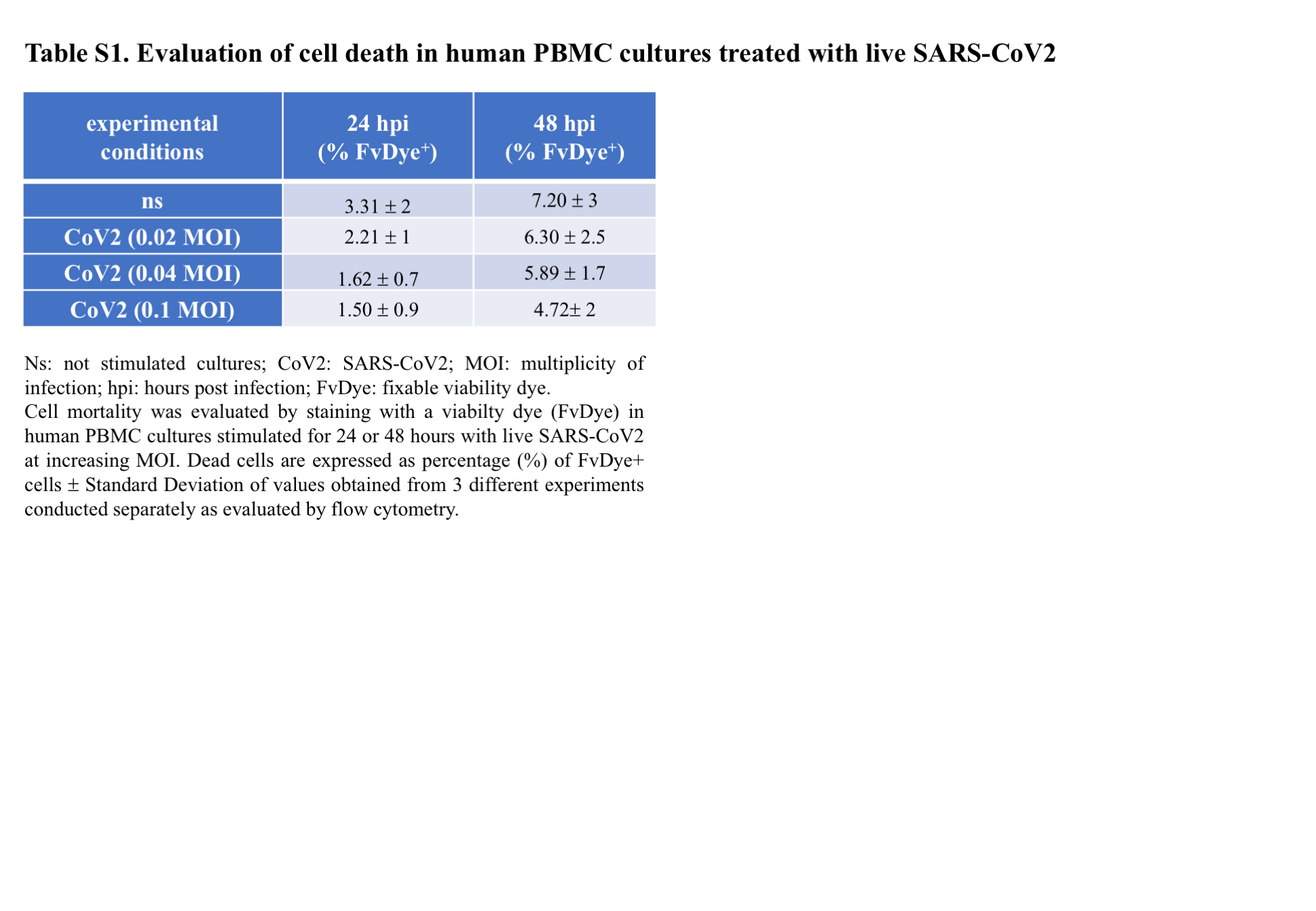

Supplement: S1 Table — (TIFF) [file ppat.1009878.s007.tiff]

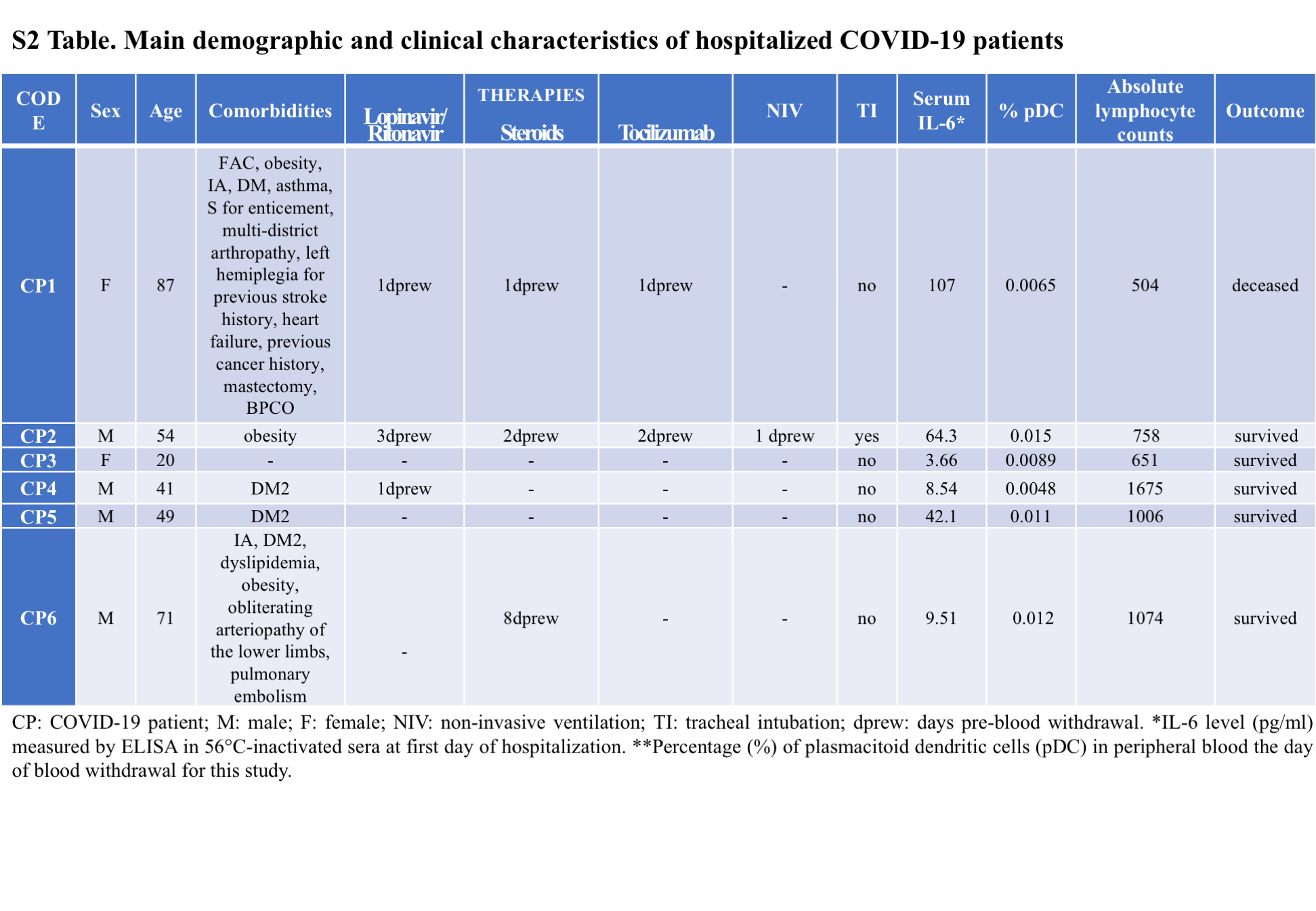

Supplement: S2 Table — (TIFF) [file ppat.1009878.s008.tiff]

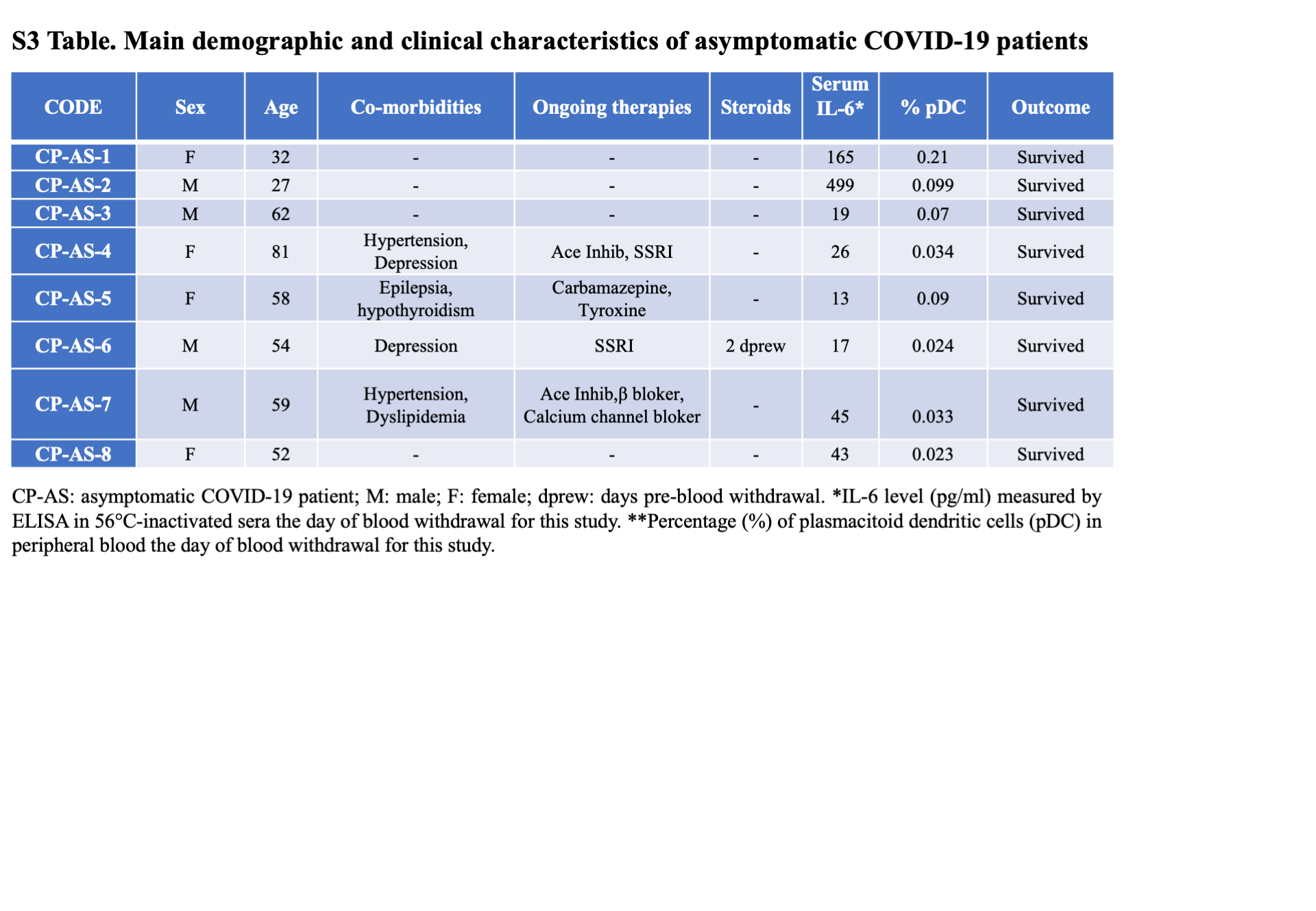

Supplement: S3 Table — (TIFF) [file ppat.1009878.s009.tiff]
