# Supplementary material for: Differential plasmacytoid dendritic cell phenotype and type I Interferon response in asymptomatic and severe COVID-19 infection
Source: PLoS Pathog. 2021 Sep 2;17(9):e1009878. doi: 10.1371/journal.ppat.1009878 (PMC8412261; doi:10.1371/journal.ppat.1009878)
Supplement: Attachment — Submitted filename: Point by point_PPATHOGENS-D-21-00780.pdf [file ppat.1009878.s010.pdf]

Point-by-point Response (PPATHOGENS-D-21-00780)

Reviewer #1

There are a couple of points that should be experimentally addressed:
a) One of the most interesting aspects of the study is that UV-inactivated Covid-19 does not stimulate production of IFN-a by PBMCs. This begs the question what about isolated pDCs? If purified pDCs also do not respond to UV inactivated Covid-19 (differently from what observed for influenza virus and HSV), it would maybe suggest the requirement for a nonstructural viral protein for induction of IFN-I. A similar phenomenon was observed for Dengue type 2 virus (Pichyangkul et. Al. JI. 2003).

We thank the reviewer for her/his comments that prompted us to further analyze the effects induced by UV inactivated SARS-CoV-2. We prepared a new viral stock for UV inactivation to make sure that UV-inactivated virus would always behave similarly. Also by using this new UV-inactivated SARS-CoV-2 stock a significant reduction in cytokine and chemokine release from whole PBMC was observed confirming data reported in the previous version of the manuscript. Consistently with these data, also in isolated pDC IFN-α production was significantly reduced upon stimulation with UV-SARS-CoV-2. The novel results derived from isolated pDC are now present in new Figure 4A, while data derived from PBMC were moved to new Supplementary Figure 4.

B) pDCs express neuropilin1 (BDCA-4), which was recently implicated in cell-entry of Covid-19. Does blockade of neuropilin1 reduce IFN-I production by pDCs?

As suggested by this reviewer, Neuropilin1 was blocked by the addition of specific monoclonal Ab to isolated pDC before stimulation with SARS-CoV-2. A dose dependent inhibition of type I IFN release was observed when the anti-Neuropilin1 Ab was used, while no difference was found in presence of anti-human IgG , thus indicating a role of this molecule in facilitating entry of virus particles in pDC. These results are inserted in new Figure 4C.

Reviewer #2:

1. The introduction should be more comprehensive about published work on pDC in COVID-19. This is the main topic of the study. Several references are cited by the authors, but the associated work is not presented in the introduction: in particular Refs 39, 45, and 47-49. Authors should also introduce pDC response to other coronaviruses, which forms important background information: refs 42 and 44. On the contrary, other parts of the introduction appear less relevant to the present study and could be moved to discussion.

Both introduction and discussion sessions were changed according to the reviewer's suggestions. Furthermore, new references on the manuscript topic were added and can be found highlighted in yellow in the tracked version of the revised manuscript.

2. In Figures 2, 3, and 6, important controls are missing. The TLR7/8 inhibitor should be used in the presence of R848 in order to show efficient inhibition (positive control). Control inhibitor (negative control: non-effective compound of similar chemical nature), should be used in comparison to the effective inhibitor for each viral titer. The authors introduce Mock as medium only (Fig legend: "Mock medium only »). This is not the usual meaning for Mock. If the condition is Medium only, it should be marked as such on the figure and its legend.

The requested control experiments were performed on both PBMC and isolated pDC and results are now inserted in the new Supplementary Figure 2 (see also description on pages 5 and 6, lanes 133-139 and on page 8, lanes 186-187 of revised manuscript). An oligonucleotide with an unrelated sequence was also used as internal control. In addition, to verify the specificity of TLR7/8 inhibitors Flu virus, a well-known TLR7 agonist, was also used to stimulate PBMC culture and then the effects of the inhibitor were analyzed as reduction of IFN-α release.

Mock description was present in the Methods section ("Cell stimulation and supernatant collection"). Nonetheless, we further detailed its meaning in Methods (see on page 21, lanes 488-490 of revised manuscript) and Results section (see on page 5, lanes 127-128). We also changed throughout all the figure legends from "mock medium only" to "mock-treated cells".

Minor Issues

Reviewer #2

1. Figure 1 presents descriptive negative results and should be shown as supplementary figure, unless the authors provide mechanistic insight.

Done. Data are now inserted in new Supplementary Figure 1.

2. Figure 4 legend mentions « in presence or absence of a 30 minute pre-treatment with a specific TLR7/8 inhibitor (I-TLR7/8, 1µM) ». However, this is not visible on the actual figure. There is no culture condition with the inhibitor.

We thank the reviewer for highlighting this mislabeling. By the way, the figure data present in old Figure 4 were moved to new Supplementary Figure 4 and the corresponding figure legend was corrected.

3. Results presented in figure 5 are completely expected given the antiviral functions of type I interferons. It could be moved to supplementary.

We agree with the reviewer on the fact that some of the data shown in old Figure 5 are somehow expected. However, collectively these results provide a proof of evidence on the presence of biologically active type I IFN released in the supernatants of PBMC stimulated with SARS-CoV-2, that is able to promote in CALU-3 cells both the expression of MxA as well as a reduction of virus replication. Based on this we would prefer to leave this figure in the main body of the manuscript as new Figure 3. We hope that the reviewer agrees.

4. In figure 6D, it is very surprising that the inhibitor induces TNF and IL-6 production by pDC. Could authors coment on that ? Is the figure just mislabled ?

The effects induced by TLR7/8 inhibitor reported in old Figure 6C (new Figure 5C) reflect the well-known cross-regulation between type I IFN release and the production of maturation-related cytokines TNF-α and IL-6 previously described by Palucka and collaborators (ref. 59 of the revised manuscript). The description of this effect was already present in the discussion section of the original manuscript (now on page 15, lanes 364-369). Interestingly, in presence of TLR7/8 inhibitor, a profound change in maturation marker profile occurs in isolated pDCs. In particular, mirroring the

switch from type I IFN-α- to TNF-α- and IL-6-producing cells, in the presence of the TLR-7/8 inhibitor the P1 phenotype induced by SARS-CoV-2 stimulation in pDC reverted into the more mature/adaptive P2 and P3 populations, exemplified by the induction of the co-stimulatory marker CD86 (new Figure 5B). Similar data were recently obtained by our group in pDC stimulated with another single-stranded RNA virus, Tick-borne encephalitis virus, where the treatment with a TLR7/8 inhibitor blocking type I IFN production enhances TNF-α and IL-6 production (also this aspect is now discussed on page 15, lanes 366-369 and new Ref. 60).

5. In Figure 8 : authors should depict individual patient/donnor results in each graph (as in 8A), instead of histograms.

Old Figure 8 (now Figure 7) was revised according to the reviewer's suggestion.

6. In Figure 8, IL-10 levels are actually very low in all conditions. Are these levels and differences biologically meaningful? What is the sensitivity limit of the assay? (should be shown on the graph)

We agree with the reviewer on the IL-10 levels found in COVID-19 patient sera are low (now in new Figure 7C). However, these data are in line with values previously published by several groups showing a direct correlation between IL-10 increase and COVID-19 severity (just a couple of papers on the topic: doi: 10.1172/JCI137244; doi: 10.1080/22221751.2020.1770129). Agreeing with the reviewer's concern on the limit of assay sensitivity, we verified this parameter, which resulted to be 3.3 pg/ml according to the datasheet of the product, thus being below the values found in the analyzed sera. Moreover, while performing the CBA assay we always consider only values within the standard curves, never those falling below.
